# Safe Transition to Pembrolizumab following Ipilimumab-Induced Guillain-Barré Syndrome: A Case Report and Review of the Literature

**DOI:** 10.1155/2019/5490707

**Published:** 2019-11-22

**Authors:** Nicholas Gravbrot, Katalin Scherer, Srinath Sundararajan

**Affiliations:** ^1^Division of Hematology-Oncology, Department of Medicine, University of Arizona Cancer Center, 1515 N. Campbell Ave., Tucson, AZ 85724-5024, USA; ^2^Department of Neurology, University of Arizona, 1501 N. Campbell Ave., Tucson, AZ 85724-5023, USA

## Abstract

**Background:**

Immune checkpoint inhibitors are novel therapies with indications for treating several solid cancers. They are associated with immune-related adverse events, which are generally well tolerated. Though rare, severe side effects may be life-threatening. One such adverse event is Guillain-Barré syndrome, which requires cessation of the immunotherapy and intravenous immunoglobulin and/or high-dose steroids to treat. No recommendations have been published regarding restarting cancer treatment after development of immunotherapy-induced Guillain-Barré syndrome.

**Case Presentation:**

A 71-year-old gentleman with recurrent, stage IIIB melanoma was started on ipilimumab (cytotoxic T lymphocyte antigen-4 inhibitor) for adjuvant treatment following radical neck dissection and radiation therapy. After completing his third cycle of ipilimumab, he developed rapidly progressive ascending paralysis. He was diagnosed with ipilimumab-induced atypical Guillain-Barré syndrome and was treated with intravenous immunoglobulin and corticosteroids. Ipilimumab was discontinued, and the patient was monitored via surveillance imaging, as there was no evidence of active disease at that time. Several months later, he was found to have recurrent disease involving the lung, requiring right lower lobectomy. Restaging revealed thoracic lymph node involvement, and he was then started on pembrolizumab (programmed cell death protein-1 inhibitor). He experienced a complete tumoral response to pembrolizumab, and he tolerated treatment well without recurrent weakness.

**Conclusions:**

Guillain-Barré syndrome is a rare but severe complication associated with immunotherapy. Our findings suggest that in patients with a history of ipilimumab-induced Guillain-Barré syndrome, pembrolizumab may possibly be a safe and effective alternative for cancer therapy.

## 1. Background

Immune checkpoint inhibitors are novel therapies indicated in the treatment of several solid tumors, most notably advanced and metastatic melanoma. Ipilimumab, a recombinant human monoclonal antibody directed against cytotoxic T lymphocyte antigen-4 (CTLA-4), blocks the central downregulatory activity of the CTLA-4/B7 axis, thus preventing T cell inactivation and indirectly upregulating T cell activity [1]. The programmed cell death protein-1 (PD-1) humanized monoclonal antibody pembrolizumab works in a similar fashion, preventing T cell suppression by blocking the peripheral interaction of PD-1 with its ligand, programmed cell death ligand-1 (PD-L1). Both therapies, in turn, facilitate an enhanced immune response against susceptible cancer cells, offering a robust and durable antitumor immunity [2, 3]. Because of their similar mechanisms of action, both CTLA-4 and PD-1 inhibitors share related adverse effects, known as immune-related adverse events (irAE). In general, these are mild and well tolerated. Common irAEs include dermatitis, enterocolitis, myalgias, arthralgias, hypothyroidism, and hypopituitarism [4–8]. Less commonly, hepatic, pulmonary, adrenal, cardiovascular, renal, pancreatic, and neurologic toxicities have been reported [6, 8–13]. Guillain-Barré syndrome (GBS) is a particularly rare neurologic irAE, with only a handful of cases reported in the literature, often with varying clinical features [3, 5, 14–20].

We present the case of a 71-year-old male who developed atypical GBS after completing his third cycle of ipilimumab. He was safely transitioned to pembrolizumab over 1 year later for recurrent disease. To our knowledge, this is the first case presented addressing the safety of initiating PD-1 inhibition following evidence of ipilimumab-induced atypical GBS.

## 2. Case Presentation

A 71-year-old gentleman with history of stage IIC left postauricular melanoma treated surgically in August 2013 developed a new left-sided preauricular mass in September 2016. Excision and sentinel node biopsy confirmed recurrent melanoma with positive nodal involvement. He subsequently underwent a modified radical neck dissection, and 1 of 29 lymph nodes was positive for metastatic disease. He was restaged with stage IIIB disease and was initially treated with adjuvant external beam radiation (48 Gy in 20 fractions) between December 2016 and January 2017. He was then enrolled in the SWOG 1404 trial and randomized to the ipilimumab arm; first treatment under protocol was in March 2017. Cycles occurred every 3 weeks; cycles 1 and 2 were tolerated well. Less than 1 week after completing cycle 3, he developed severe, progressive, symmetric ascending weakness without sensory loss. Over the course of several days, the paralysis progressed to inability to stand and arm weakness. There was no dysphagia, ptosis, neck weakness, or respiratory involvement. Neurological examination showed profound, symmetrical, proximal greater than distal upper and lower extremity weakness and unobtainable deep tendon reflexes. The patient eventually developed mild dysphagia and shortness of breath but never required intubation.

The patient was admitted for workup and treatment. Complete blood count and comprehensive metabolic panel were within normal limits. Magnetic resonance imaging (MRI) of the spine was not possible due to the presence of a spinal cord stimulator for chronic low back and radicular pain. Computed tomography (CT) of the total spine and brain showed no abnormalities. Cerebrospinal fluid (CSF) analysis was normal 11 days after the 3^rd^ dose of ipilimumab (6 days after the onset of weakness). Creatine phosphokinase, aldolase, lactate dehydrogenase, and serum protein electrophoresis were unremarkable. Thyroid function tests and cortisol were within normal limits. C-reactive protein and erythrocyte sedimentation rate were elevated to 0.71 mg/dL and 121 mm/hr, respectively.

Given his clinical and laboratory findings, the patient was diagnosed with atypical GBS secondary to ipilimumab therapy. Intravenous immunoglobulin (IVIG) was started day 11 post ipilimumab (day 6 of weakness), and a 2 g/kg total dose was completed over 5 days. Prednisone was started at 30 mg daily in divided doses at the same time. He improved rapidly. Upon admission, he was bedbound; upon discharge from the rehabilitation hospital 3 weeks later, he was ambulatory with a walker; and 6 weeks post onset of paralysis, he was ambulatory with a cane. Electromyography and nerve conduction studies were performed 6 weeks after the onset of paralysis and confirmed sequela of an acute sensorimotor polyradiculoneuropathy with mixed axonal/demyelinating features consistent with GBS. There was significant active denervation and reinnervation in distal upper and lower limb muscles. Sensory and motor nerve velocities were mildly reduced, with a length-dependent loss of amplitudes. There was no temporal dispersion, and no conduction blocks were found. Repetitive nerve stimulation was normal. Steroids were continued for a few months and tapered. His strength returned close to baseline after several months, with his last neurological exam documenting mild proximal muscle weakness, areflexia, and slightly unsteady but unassisted gait 3 months after onset of GBS. The patient was not restarted on ipilimumab and opted for surveillance, as scans showed no evidence of disease.

The patient was followed with serial CT scans of the chest, abdomen, and pelvis. A 2 cm right lower lung nodule was noted in February 2018; the biopsy showed recurrent melanoma. He then underwent right lower lobectomy with complete lymphadenectomy in May 2018. Bronchovascular margins were negative, but 3 lymph nodes had melanoma involvement. Molecular profiling showed that the patient did not have a BRAF V600 domain mutation, and the tumor had a high PD-L1 expression (50%) and mutational burden. Restaging with positron emission tomography (PET)/CT within a few months revealed additional left paratracheal lymph node invasion. The patient was then started on anti-PD-1 therapy with pembrolizumab in June 2018, with special attention given toward close monitoring for any neurologic complications. The patient tolerated this regimen remarkably well, and repeat PET/CT in September 2018 (after cycle 4) demonstrated complete response to treatment, with no evidence of disease. Today, the patient continues on maintenance pembrolizumab and recently completed his 15^th^ cycle. There has been no evidence of recurrent neurologic symptoms over the course of his treatment, and his most recent PET/CT in March 2019 showed continued complete response to therapy.

## 3. Discussion

Neurologic irAEs are uncommonly reported but potentially serious complications of checkpoint inhibitor use. In a recent article, Garcia et al. [3] reviewed the literature on the heterogeneity of ipilimumab-associated neurologic irAEs; manifestations included myasthenia gravis, Bell's palsy, meningitis, hypophysitis, meningo-radiculo-neuritis, acute cerebellitis, transverse myelitis, and GBS, as in our patient. Kao et al. [21] described a similar range of neurologic irAEs associated with PD-1 inhibitors. While the exact mechanism underlying these complications has not been elucidated, multiple hypotheses have been proposed: loss of peripheral tolerance, T regulatory cell deficit (both quantitative and qualitative), and molecular mimicry between malignant melanocytes and other neural crest derivatives (Schwann cells, for instance) [3, 5]. Differences in human leukocyte antigen (HLA) expression may also be implicated, but there is insufficient evidence at this point to clearly define an association with irAE incidence [3].

GBS is a particularly concerning neurologic condition, with the potential to progress to life-threatening weakness and dysautonomia. Classically, GBS develops in response to known or unknown immunological stimulus, such as *Campylobacter* or viral infection, surgery, immunizations, or trauma, but several medications, including immune checkpoint inhibitors, have been known to induce the disease. Clinical features of GBS are heterogenous, and multiple variants have been described. Classic GBS, or the acute inflammatory demyelinating polyneuropathy (AIDP) variant, presents with rapidly progressive, symmetric, ascending weakness of the upper and lower extremities, with loss of deep tendon reflexes and variable sensory deficit. In severe cases, bulbar muscle and diaphragmatic involvement may be seen; the latter places the patient at significant risk for respiratory failure, often necessitating intubation. Other variants include pure motor neuropathy, pure sensory neuropathy, cranial nerve palsy, pharyngeal-cervical-brachial palsy, occlusive enteric neuropathy, and pandysautonomia [5, 15, 16, 22].

The literature on checkpoint inhibitor-induced GBS is limited, as only a small number of cases have been reported, summarized in Table 1. Both CTLA-4 and PD-1 inhibitors have been implicated [3, 5, 14–20, 23–27]. Multiple GBS variants have been described, including classic AIDP, enteric neuropathy, and pandysautonomia [3, 5, 14–20]. Treatment has consisted of corticosteroids and/or IVIG, as well as discontinuation of the implicated checkpoint inhibitor. Plasmapheresis and other immunomodulatory therapies such as tacrolimus have been added in refractory disease [16, 19]. In the case of pandysautonomia, pressors, intubation, indwelling urinary catheter, and total parenteral nutrition were also required for supportive care [5]. In general, long-term outcomes have been excellent, though select patients have expired from GBS despite aggressive intervention [15, 16, 19, 23].

Understandably, given the small number of cases, there is a paucity of literature specifically discussing reinitiation of cancer-specific therapy following checkpoint inhibitor-induced GBS. The safety of transitioning to PD-1 inhibitors in patients with history of major toxicity with CTLA-4 inhibition was explored previously by Menzies et al., though no cases of ipilimumab-related GBS were mentioned [28]. It was found that across multiple domains including other neurologic AEs, anti-PD-1 therapies were well tolerated and overall safe to use in patients with history of significant ipilimumab toxicity [28]. In our case, we extrapolated that the results of this study may extend to ipilimumab-induced GBS, though we proceeded with caution, especially considering pembrolizumab's independent association with GBS, maintaining a low threshold for evaluation and treatment with any new or recurrent neurologic change. Thus far, it would appear that the transition to pembrolizumab was not only safe but also effective in treating his metastatic melanoma.

To our knowledge, our patient is the first reported case of safely transitioning to pembrolizumab following ipilimumab-related GBS. This, coupled with Menzies et al.'s broader findings [28], may suggest that use of PD-1 inhibitors is a safe alternative for patients whose cancer progresses after discontinuing CTLA-4-directed therapy for drug-induced GBS. However, no definitive conclusions can be drawn from case reports or case series, as we would need more safety data. For now, we recommend maintaining a high clinical suspicion for recurrent drug-induced GBS should any new or recurrent neurologic symptoms develop while on anti-PD-1 therapy. Patients should also be educated about the varying presentations of GBS so that they understand the risk and may be able to better recognize early symptoms, therefore seeking prompt medical evaluation and intervention when necessary.

## 4. Conclusion

Anti-CTLA-4 and anti-PD-1/PD-L1 therapies are useful in treating several types of cancer, and though they are generally well tolerated, they are associated with infrequent, severe irAEs. GBS is one such rare complication with diverse and potentially fatal manifestations. To date, there are no published recommendations about restarting different immunotherapies in the context of immunotherapy-induced GBS. Herein, we discuss the case of a 71-year-old gentleman who developed GBS in association with ipilimumab and was found to have progression of melanoma several months after stopping the CTLA-4 inhibitor. Anti-PD-1 therapy was subsequently initiated and tolerated exceptionally well, with no evidence of neurologic deficit. Complete melanoma response was achieved after only a few cycles of therapy. These findings may suggest that PD-1 inhibitors are safe and effective to use in patients with ipilimumab-induced GBS, though additional studies with more patients are necessary to evaluate this further.

## Figures and Tables

**Table 1:** Case reports/series of checkpoint inhibitor-induced GBS.

Cases	Checkpoint inhibitor	GBS variant	Treatment	Outcome
Wilgenhof and Neyns [14]	Ipilimumab/CTLA-4	AIDP	Corticosteroids	Recovery
Bot et al. [15]	Ipilimumab/CTLA-4	AIDP	IVIG	Death
Gaudy-Marqueste et al. [16]	Ipilimumab/CTLA-4	Enteric neuropathy	Corticosteroids, anti-TNF therapy, tacrolimus, plasmapheresis, orogastric drainage, and ventilatory assistance	Death
Jacob et al. [23]	Nivolumab/PD-1	AIDP	IVIG, plasmapheresis, and intubation	Death
Patel et al. [17]	Ipilimumab/CTLA-4	AIDP	Corticosteroids, IVIG	Recovery
Supakornnumporn and Katirji [18]	Combination ipilimumab+nivolumab	AIDP	Corticosteroids, IVIG, and nasogastric tube	Recovery
Wu et al. [5]	Ipilimumab/CTLA-4	Pandysautonomia	Corticosteroids, IVIG, pressors, intubation, indwelling urinary catheter, and TPN	Recovery
Fukumoto et al. [24]	Nivolumab/PD-1	AIDP	Corticosteroids, IVIG	Recovery
Garcia et al. [3]	Ipilimumab/CTLA-4	AIDP	Corticosteroids	Recovery
Kyriazoglou et al. [27]	Nivolumab/PD-1	AIDP	Corticosteroids, IVIG	Recovery
Manam et al.—2 cases [19]	Pembrolizumab/PD-1 ×2	(1) AIDP (2) AIDP	(1) Corticosteroids, IVIG, and plasmapheresis (2) Corticosteroids, IVIG, intubation, and plasmapheresis	(1) Recovery (2) Death
Nukui et al. [25]	Nivolumab/PD-1	AIDP	Corticosteroids, IVIG	Recovery
Ong et al. [20]	Pembrolizumab/PD-1	AIDP	Corticosteroids, IVIG	Recovery
Thapa et al. [26]	Nivolumab/PD-1	AIDP	Corticosteroids, IVIG, and intubation	Prevention of disease progression

AIDP = acute inflammatory demyelinating polyneuropathy; IVIG = intravenous immunoglobulin; TNF = tissue necrosis factor; TPN = total parenteral nutrition.

## Abbreviations

AIDP: Acute inflammatory demyelinating polyneuropathy
AEs: Adverse events
CSF: Cerebrospinal fluid
CT: Computed tomography
CTLA-4: Cytotoxic T lymphocyte antigen-4
GBS: Guillain-Barré syndrome
irAE: Immune-related adverse events
MRI: Magnetic resonance imaging
PET: Positron emission tomography
PD-1: Programmed cell death protein-1
PD-L1: Programmed cell death protein-1 ligand.
